# Enhanced biogeochemical remediation of Pb-contaminated loess *via* MICP integrated with graphene nanomaterials

**DOI:** 10.1039/d5ra04818d

**Published:** 2025-08-18

**Authors:** Xinwen Wang, Shixu Zhang, Ke Chen

**Affiliations:** a School of Intelligent Construction and Civil Engineering, Luoyang Institute of Science and Technology Luoyang 471023 China 200900600391@lit.edu.cn; b School of Civil Engineering, Xi'an University of Architecture and Technology Xi'an 710055 China zhangsx@xauat.edu.cn; c School of Civil Engineering, Dazhou Technician College Dazhou 635001 China 200900600432@lit.edu.cn

## Abstract

This study explores the synergistic effects of microbially induced carbonate precipitation (MICP) combined with graphene-based adsorptive materials, namely graphene (GR) and graphene oxide (GO), for the remediation of lead-contaminated loess. A series of systematic experiments were conducted, including unconfined compressive strength (UCS) testing, toxicity characteristic leaching procedure analysis, zeta potential measurements, scanning electron microscopy (SEM) observation, X-ray fluorescence (XRF) analysis, and microstructural modeling. The results revealed that MICP effectively improved soil strength and immobilized Pb^2+^ through carbonate precipitation and microbial surface adsorption, reducing lead leaching concentrations by up to 39.56%. The addition of GR and GO significantly enhanced the remediation performance by further lowering Pb^2+^ mobility and improving soil mechanical properties. Optimal results were achieved with 1.0% GO content, where UCS increased by approximately 11.7% compared to MICP alone, and lead leaching concentration was reduced by 61.63% relative to untreated soil. Microstructural analysis indicated that the combined remediation process promoted denser soil packing, enhanced calcium carbonate distribution, and facilitated multi-pathway Pb^2+^ immobilization, including precipitation, chemical adsorption, and physical encapsulation. GO exhibited superior performance due to its higher negative surface charge, larger specific surface area, and abundant oxygen-containing functional groups. These findings highlight the potential of integrating MICP with graphene-based materials for the simultaneous stabilization and strengthening of heavy metal-contaminated loess, providing valuable insights for the development of advanced soil remediation technologies.

## Abstract 2

This study explores the synergistic effects of microbially induced carbonate precipitation (MICP) combined with graphene-based adsorptive materials, namely graphene (GR) and graphene oxide (GO), for the remediation of lead-contaminated loess.

## Graphical abstract

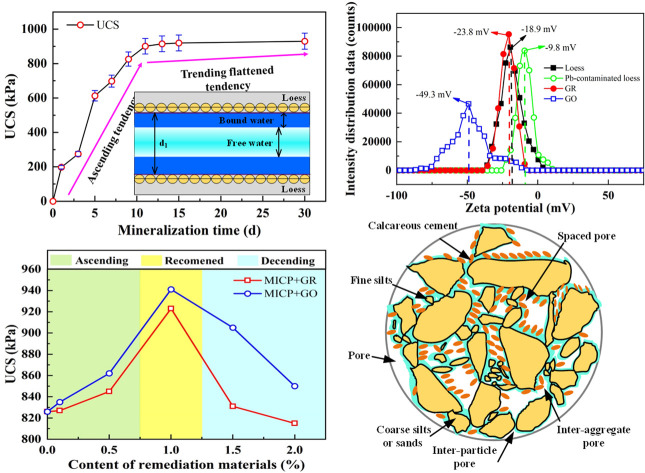

## 1. Introduction

The accelerated pace of industrial and technological development in recent years has resulted in the pervasive contamination of soils with heavy metals and metalloids.^1,2^ Heavy metals, recognized as persistent and toxic pollutants, pose substantial risks to plant and animal life, as well as to human health.^3,4^ Consequently, soil contamination by heavy metals has emerged as a critical global environmental issue, demanding immediate and effective remediation strategies.^5,7^ However, the intricate chemical speciation, high bioaccumulation potential, latent toxicity, and environmental persistence of heavy metals continue to hinder global remediation efforts.^8,9^ Current remediation strategies for heavy metal-contaminated soils are broadly classified into three categories: physical, chemical, and biological approaches.^8–10^ The distinctive physical and chemical properties of loess—marked by high porosity, strong permeability, and notable alkalinity (pH 8.5–9.0)—present considerable challenges for the implementation of conventional remediation techniques.^11–13^ Physical methods (*e.g.*, soil replacement) are economically prohibitive and compromise the structural integrity of the soil; chemical approaches (*e.g.*, phosphate stabilization) often lead to the formation of soluble lead complexes under alkaline conditions; and biological methods (*e.g.*, phytoremediation) are constrained by the arid climate and limited biomass characteristic of loess regions. While each of these methods holds potential, their practical application remains constrained by inherent limitations and suboptimal outcomes, preventing them from gaining universal acceptance.^14–17^ Although extensive research has been conducted on the remediation of complexly contaminated soils, investigations specifically targeting lead-contaminated loess remain notably limited.

Microbial remediation of mildly contaminated soils has emerged as an environmentally benign and sustainable technology, and in recent years it has been increasingly applied to the treatment of heavy metal-contaminated soils.^18–20^ The pathways involved in such remediation include denitrification, methane oxidation, sulfate reduction, and urea hydrolysis. Among these, urea hydrolysis-based techniques have garnered significant attention due to their rapid activation and their relevance to the Microbially Induced Calcite Precipitation (MICP) process for immobilizing heavy metals.^21,22^ The MICP mechanism involves urease-producing bacteria hydrolyzing urea to generate carbonate ions (CO_3_^2−^), which subsequently react biochemically with calcium ions (Ca^2+^) to form calcite crystals. Within this process, heavy metal ions may be immobilized by substituting divalent cations (such as Ca^2+^) within the calcite lattice, forming insoluble carbonate precipitates through direct reaction with CO_3_^2−^, or becoming encapsulated within the calcite matrix *via* co-precipitation.^23^ These mechanisms effectively convert soluble heavy metals into stable, insoluble forms, achieving *in situ* immobilization. As a novel remediation technology that synergistically integrates chemical stabilization and microbial activity, MICP offers a promising approach for heavy metal stabilization and removal in contaminated soils. Current research primarily focuses on elucidating the mechanisms of MICP-mediated heavy metal solidification and assessing the environmental safety of the treated soils. The interaction between MICP and heavy metals is influenced by various factors, including the type and concentration of heavy metals, soil characteristics, and bacterial strains employed. A substantial body of literature affirms the applicability of MICP in sealing porous media, reinforcing stone and cement-based materials, and repairing surface cracks and defects. With regard to the stabilization of heavy metal-contaminated soils, numerous studies have demonstrated the efficacy of MICP in immobilizing exchangeable heavy metal ions by inducing mineralization and transforming them into carbonate-bound forms. For instance, Li *et al.*^19^ applied MICP to lead- and zinc-contaminated soils, reporting reductions in leachable lead and zinc concentrations by 44.81% and 46.19%, respectively, after a 10 days mineralization period. Kang *et al.*^24^ employed a consortium of four ureolytic bacterial strains to achieve microbial mineralization in sand columns contaminated with multiple metals including copper, lead, and cadmium, attaining removal efficiencies ranging from 45.4% to 98.5%. Sharmam *et al.*^25^ found that the fixation rates for lead, zinc, and chromium exceeded 92%, although zinc showed relatively lower stabilization. Chen *et al.*^26^ using an *in situ* activation approach, reduced the water-soluble exchangeable fraction of copper in soil from 45.54 mg kg^−1^ to 1.55 mg kg^−1^. Building on these findings, further investigation using 0.5 mol L^−1^ Ca^2+^ was conducted to elucidate the immobilization mechanism of cadmium in contaminated soils. Extensive research has been conducted on the use of individual adsorptive materials or biological methods for the remediation of heavy metal-contaminated soils. However, studies integrating MICP technology with adsorptive materials to simultaneously reduce heavy metal leachability and enhance the mechanical properties of the stabilized soils remain remarkably scarce.

Although Microbially Induced Carbonate Precipitation (MICP) has demonstrated considerable potential in encapsulating heavy metals *via* the formation of carbonate minerals, its application in loess remains limited by the high proportion of exchangeable lead species (>40%) and the relatively low solidification efficiency (<70%), as well as concerns regarding long-term stability.^27–32^ Recent studies have revealed that graphene-based materials, owing to their abundance of oxygen-containing functional groups (–COOH/–OH), can effectively chelate heavy metal ions.^33–35^ However, adsorption alone often fails to achieve permanent immobilization of lead. In light of these challenges, this study proposes a combined remediation strategy integrating MICP with graphene materials for the solidification/stabilization of lead-contaminated loess. The efficacy of this approach is evaluated through unconfined compressive strength tests and toxicity leaching assessments, examining both the mechanical reinforcement and heavy metal stabilization effects. Furthermore, the underlying mechanisms are elucidated using zeta potential analysis, scanning electron microscopy (SEM), and X-ray fluorescence (XRF) testing. The findings provide theoretical insight and practical guidance for the development of enhanced MICP-based remediation strategies, highlighting the potential of graphene-assisted MICP to effectively reduce lead leachability and improve the structural integrity of contaminated loess.

## 2. Materials and methods

### 2.1. Testing materials

#### 2.1.1. Tested soil

**Fig. 1:**
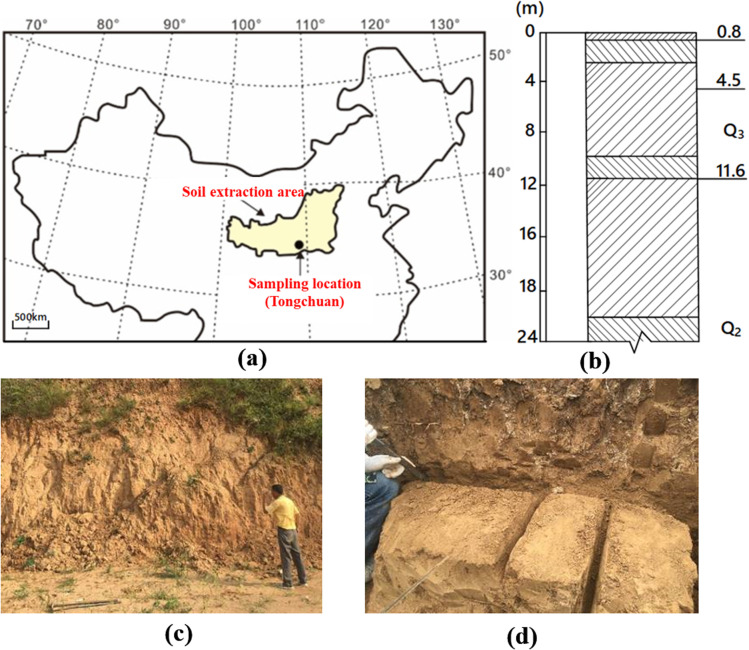
(a) Geographical location, (b) stratigraphic profile, and (c and d) field sampling of loess in the Lantian area.

The Guanzhong region, situated in the middle reaches of the Yellow River, is among the largest and most representative loess deposition zones worldwide, encompassing a total area of approximately 6.4 × 10^5^ km^2^. Fig. 1a illustrates the sampling location of loess utilized in this study, located in Tongchuan County, Xi'an City, Shaanxi Province. This site lies within a typical profile area of the central Loess Plateau, characterized by abundant soil sources and strong representativeness. The region experiences a warm temperate, semi-arid to semi-humid climate, and features classical aeolian loess accumulation. The prevailing geomorphological types are loess tablelands and ridges. Fig. 1b presents the stratigraphic profile of the study area, which is primarily composed of the Upper and Middle Pleistocene series (Q_3_ and Q_2_). The Q_3_ loess (third stage of Quaternary loess) layer is approximately 11.6 meters thick, with stratification from top to bottom including a cultivated soil layer (about 0.8 m), a silt loess layer, and a paleosol layer, all exhibiting distinct depositional structures and well-defined bedding.

**Fig. 2:**
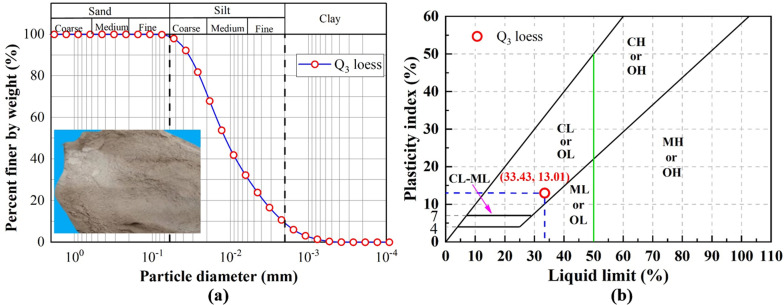
(a) Particle size distribution of Q_3_ loess and (b) liquid limit and plastic index.

The loess samples in this study were collected from a depth of 0.5 to 1.0 meters below the surface (Fig.1a and b). As shown in Fig. 1c and d, undisturbed, exposed sections of the natural loess profile were selected for sampling. Using shovels and cutting tools, regular-shaped soil blocks were extracted, sealed, and transported to the laboratory. The collected loess appears light grayish-yellow, with a loose texture, dry feel, and no evident cementation. Following the standard pretreatment protocols established by Xu *et al.*^36,37^ and Hou *et al.*^38^ the specimens were air-dried, ground, and sieved before undergoing particle size analysis using a Malvern Mastersizer 2000 laser granulometer. The particle size distribution curve of Q_3_ loess is shown in Fig. 2a. The Q_3_ loess consists of approximately 94.16% fines and 5.84% sands. Further, the liquid limit and plasticity index of Q_3_ loess are 32.52% and 18.68% respectively. Based upon the liquid limit and plasticity index (Fig. 2b), the Q_3_ loess is classed as the low plasticity clay (CL). The physical properties of Q_3_ loess are summarised in Table 1. The results revealed that the loess is predominantly composed of silt, with smaller fractions of clay and sand. According to the ASTM standards (2011),^39^ the loess is categorized as low-plasticity clay (CL). The key physical and mechanical properties listed in Table 1 include liquid limit, plastic limit, specific gravity, optimum moisture content and dry density. These parameters collectively highlight the engineering suitability and hydraulic sensitivity of the loess in this region.

**Table 1:** Physicochemical properties of the loess

Physical index	Data
Fines (%)	94.16
Sand (%)	5.84
Gravel (%)	0
Specific gravity, Gs	2.70
Void ratio, *e*	0.86
Dry density, *ρ*_dmax_/(g cm^−3^)	1.73
Initial water content, (%)	16.6
The Atterberg limit	
Liquid limit, (%)	32.52
Plastic limit, (%)	18.68
Soil classification	CL

#### 2.1.2. Bacteria and cementation solutions for MICP treatment

**Fig. 3:**
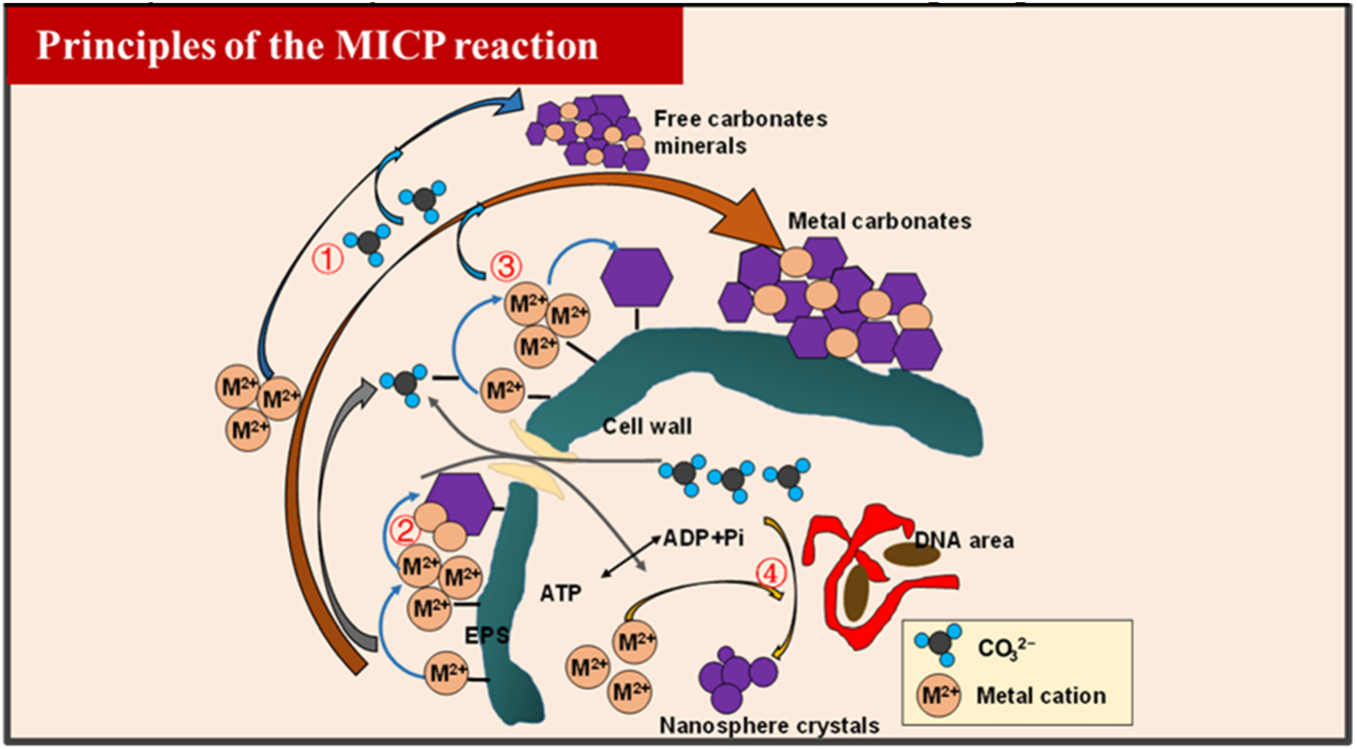
The process of MICP (EPS: extracellular polymeric substances) (modified from He *et al.*^6^).

Microbially Induced Carbonate Precipitation (MICP) is an emerging biomineralization technique that leverages specific microbial metabolic processes to improve soil mechanical behavior by fostering the *in situ* generation of calcium carbonate, which subsequently binds soil particles into aggregated structures.^40–42^ The microorganisms involved in this process are typically ureolytic and alkaliphilic in nature, including strains such as *Bacillus pasteurii*, *Bacillus sphaericus*, *Bacillus licheniformis*, and certain nitrate-reducing bacteria, which are capable of surviving in high-pH environments and catalyzing urea hydrolysis (see Fig. 3). Among these, *Bacillus pasteurii* has been extensively studied due to its robustness under various environmental conditions, ease of cultivation, and high urease activity that directly contributes to efficient CaCO_3_ precipitation.^43–45^

In this study, *Sporosarcina pasteurii* (CGMCC1.3687), a well-characterized ureolytic bacterium, was obtained from the China General Microbiological Culture Collection Center and utilized as the biological agent. The bacterial culture medium was prepared based on protocols established by Jiang *et al.*^46^ and Wang *et al.*,^43^ containing urea (20 g L^−1^) as the urease substrate, peptone (5 g L^−1^) and yeast extract (3 g L^−1^) as nitrogen and growth factor sources, and manganese sulfate (0.01 g L^−1^) to facilitate enzymatic activity. The pH of the medium was finely tuned to neutrality (pH 7.0) using a 10% NaOH solution prior to autoclaving at 121 °C for 20 minutes to ensure sterility.

Post-sterilization, the medium was inoculated with a 1 : 100 volumetric ratio of bacterial seed culture and incubated under aerobic conditions in a rotary shaker set to 30 °C and 180 rpm for 48 hours. Bacterial growth was quantified spectrophotometrically *via* optical density measurements at 600 nm (OD_600_ ≈ 1.70), while urease enzymatic activity was assessed through the change in electrical conductivity over a 5 minutes interval, indicating a hydrolysis rate of approximately 4.0 mM urea per min.

For the subsequent biocementation process, a cementation solution was formulated by dissolving equimolar concentrations of urea and calcium chloride in deionized water, serving as nitrogen and calcium sources respectively, in alignment with the methodology reported by Ahenkorah *et al.*^47^ To mitigate the loss of urea through volatilization, the solution was freshly prepared and applied within one hour of preparation, ensuring maximal reactivity and consistency throughout the MICP treatment phase.

#### 2.1.3. Adsorption materials

**Fig. 4:**
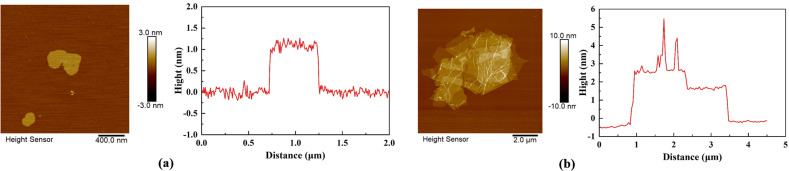
Atomic force microscopy results of graphene: (a) single-layer graphene and (b) multilayer graphene oxide.

To enhance the remediation efficiency of MICP technology for contaminated soils, two types of adsorptive additives—single-layer graphene (GR) and multilayer graphene oxide (GO)—were introduced during the mineralization process, as illustrated in Fig. 2. Fig. 4 presents the atomic force microscopy (AFM) characterization of both single-layer and multilayer graphene materials. As shown in the images, GR exhibits a highly ordered lamellar structure with a smooth and uniform surface, curled edges, and a thickness of approximately 1 nm. The GO used in this study appears veil-like, with a wrinkled and rough surface morphology, accompanied by slight folding. Its average thickness is around 3.2 nm, indicating the presence of multilayered stacking, composed of roughly three overlapping graphene sheets. The AFM images reveal surface topographies featuring grooves and pores, which are attributed to the electrostatic repulsion induced by the functional groups on the graphene surface.

### 2.2. Experimental methods

#### 2.2.1. Lead-contaminated loess preparation

The loess samples were initially oven-dried, mechanically ground, and passed through a standard sieve to ensure uniform particle size distribution prior to experimental use. To prepare the contaminant source, analytical-grade lead nitrate [Pb(NO_3_)_2_] was accurately weighed and fully dissolved in deionized water to obtain a homogeneous Pb^2+^ solution. A calculated volume of the prepared solution was evenly sprayed onto the processed loess, followed by intensive manual mixing to facilitate uniform dispersion of lead ions within the soil matrix. This procedure yielded artificially contaminated loess with a target Pb concentration of 500 mg kg^−1^. Subsequently, the treated samples were subjected to natural air-drying at ambient laboratory conditions (∼25 °C) for a duration of one month. This curing period allowed sufficient time for physicochemical interactions between the heavy metal ions and the loess particles, thereby forming what is referred to as the untreated Pb-contaminated loess, which served as the baseline material for subsequent remediation investigations.

**Fig. 5:**
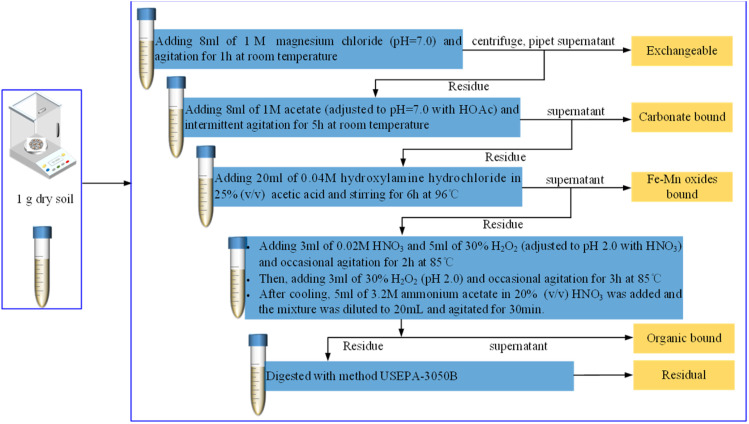
Sequential extraction procedure for Pb speciation analysis based on the modified BCR protocol.

**Fig. 6:**
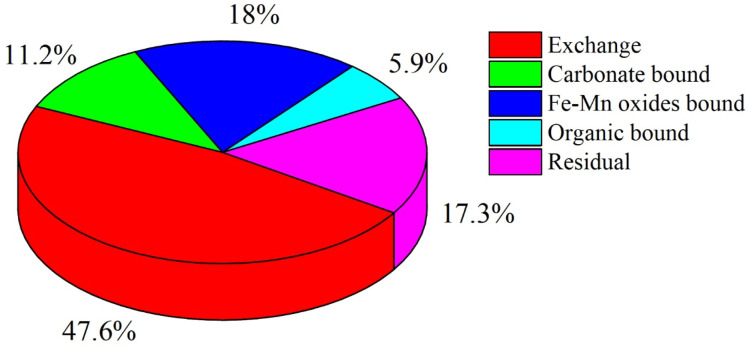
Distribution of lead chemical fractions in artificially contaminated loess.

To further characterize the chemical speciation and environmental mobility of Pb in the contaminated loess, a five-step sequential extraction procedure based on the BCR protocol (modified from USEPA 3050B) was conducted. The schematic workflow for this procedure is illustrated in Fig. 5. The Pb fractionation results are summarized in Fig. 6. The dominant fraction was the exchangeable form, accounting for 47.6% of the total Pb content, indicating high bioavailability and environmental risk. The residual fraction contributed 17.3%, while the Fe–Mn oxide-bound and carbonate-bound fractions accounted for18.0% and 11.2%, respectively. The organically bound form constituted only 5.9% of total Pb. This distribution profile suggests that nearly half of the Pb is readily mobilizable under slight environmental changes, emphasizing the need for effective remediation strategies targeting the labile fractions.

#### 2.2.2. Specimen preparation method

In the preparation of individual specimens for the solidification and stabilization of heavy metal-contaminated soils using MICP technology, the adsorptive materials were weighed according to predetermined proportions. A total of 150 g of dried soil was thoroughly mixed with 40 mL of bacterial suspension at an OD_600_ of 1.2. The well-mixed soil and bacterial solution were then placed into cylindrical flexible molds with a diameter of 40 mm and a height of 80 mm. Following this, the specimens, still encased within the molds, were subjected to a free percolation soaking method. They were fully immersed in 1 L of cementation nutrient solution, with an aeration pump inserted to facilitate sufficient mineralization reactions. Upon reaching the designated mineralization period, the specimens were carefully removed, the flexible molds detached, and the samples allowed to air-dry naturally.

Building upon the conventional MICP-based solidification and stabilization process, this method incorporates adsorptive materials to improve the remediation efficiency and enhance the overall performance of the treated soils. Two types of nano-scale adsorptive materials, GR and GO, were selected as auxiliary agents to synergize with the Microbially Induced Carbonate Precipitation (MICP) technique, aiming to enhance the solidification and stabilization of heavy metal-contaminated soils. Owing to their exceptional specific surface area, superior adsorption capacity, and abundant functional groups, GR and GO effectively regulate soil pore structure, improve the capture of heavy metal ions, and reinforce soil strength. During sample preparation, the graphene-based additives were thoroughly blended with the bacterial suspension–soil mixture according to the predetermined dosage ratios. The bacterial suspension was adjusted to an optical density (OD_600_) of 1.2, and 40 mL of this suspension was mixed with 150 g of dried loess. The designated amounts of adsorptive materials were subsequently added during the mixing process to ensure uniform dispersion. To systematically evaluate the influence of additive dosage on remediation performance, five dosage levels were established at 0.1%, 0.5%, 1.0%, 1.5%, and 2.0% by mass relative to the dry soil weight. Mechanical stirring, supplemented by manual mixing, was employed to maximize the homogeneity and dispersibility of the soil mixtures. Once thoroughly blended, the mixtures were packed into flexible cylindrical molds with a diameter of 40 mm and a height of 80 mm. Moderate tamping was applied during molding to eliminate air pockets and ensure structural compactness. The molded specimens were then immersed in containers filled with 1 L of cementation nutrient solution, where free percolation soaking was employed for mineralization. Continuous aeration was provided *via* oxygen pumps to facilitate the mineralization reactions. Upon completion of the designated mineralization period, the specimens were demolded and air-dried naturally, ready for subsequent mechanical testing and evaluation of heavy metal immobilization efficacy.

#### 2.2.3. Leaching test

To evaluate the leaching behavior of heavy metals in solidified and stabilized soil samples, this study employed Method 1311, known as the Toxicity Characteristic Leaching Procedure (TCLP), as established by the U.S. Environmental Protection Agency (EPA).^48^ Recognized as a globally adopted standardized testing method, TCLP is widely used to assess the potential toxic risks posed by waste materials under environmental conditions, particularly in evaluating the leaching potential of heavy metals during landfilling or disposal processes. It is also routinely applied to examine the effectiveness and environmental safety of hazardous waste stabilization technologies. The preparation of the TCLP extraction solution strictly followed standardized protocols. A mixture of 5.7 mL of glacial acetic acid and 64.3 mL of sodium hydroxide solution (1 N NaOH) was diluted with deionized water to a total volume of 1 L. The pH of the solution was carefully adjusted to 4.93 ± 0.05 using a calibrated pH meter, ensuring the acidic conditions required for the leaching test. The treated soil samples were air-dried and ground to pass through a 2 mm sieve to ensure uniformity. A 50 g portion of the sieved soil was placed into a 500 mL TCLP extraction vessel, to which 500 mL of the prepared extraction solution was added. The sealed extraction vessels were mounted on a mechanical rotary shaker and agitated continuously at a speed of 30 ± 2 rpm for 18 hours, simulating the long-term leaching behavior of heavy metals under soaking conditions. After agitation, the mixtures were allowed to settle, and the supernatants were filtered through membrane filters with a pore size of 0.45 μm to remove suspended solids. Finally, the concentrations of lead ions in the filtrates were quantitatively performed using a Z-8200 Polarised Zeeman Atomic Absorption Spectrometer (Hitachi) to assess the leaching potential of lead and the environmental safety of the solidified loess samples after treatment.

#### 2.2.4. Zeta potential measurement method

To systematically analyze the surface charge characteristics of graphene (GR) and graphene oxide (GO) nanomaterials, as well as to investigate the effects of lead contamination on the surface electrochemical behavior of loess particles, this study conducted zeta potential measurements on four types of samples: (1) natural loess, (2) lead-contaminated loess, (3) GR nanomaterials, and (4) GO nanomaterials.

Prior to testing, all specimens had pretreatment done as per protocol. The loess samples were first air-dried in a room at constant weight. The samples were then ground and sieved through 2 mm mesh. 0.1 g of soil was dispersed in 100 mL deionized water to obtain a suspension of 0.1 wt% (1 : 1000). In order to ensure a uniform dispersion, the suspensions were stirred for 30 minutes on a magnetic stirrer. They were also subjected to ultrasonic treatment for 10 minutes to prevent aggregation of the particles. For GR and GO nanomaterials, suspensions were similarly prepared at a concentration of 0.05 wt% in deionized water, followed by ultrasonic treatment for 30 minutes to ensure full exfoliation and dispersion, forming stable colloidal solutions. The initial pH of all suspensions was maintained at neutral (approximately pH 7.0). For studies involving pH-dependent zeta potential behavior, the pH was adjusted using dilute hydrochloric acid or sodium hydroxide solutions to achieve the desired conditions. Zeta potential measurements were performed using a Malvern Zetasizer Nano ZS 90 analyzer (Malvern Instruments, UK) based on the principle of Electrophoretic Light Scattering (ELS). The measurement temperature was kept constant at 25 °C, with refractive index and dielectric constant settings corresponding to aqueous systems. To ensure accuracy and reproducibility, each sample was measured at least three times, and the average value was recorded as the final result. If sedimentation was observed in the suspensions during testing, a brief ultrasonic treatment was applied before measurement to re-disperse the particles. By comparing the zeta potential values of natural loess, lead-contaminated loess, and GR and GO nanomaterials, this study systematically elucidates the changes in surface electrochemical properties of loess particles following lead contamination, while also assessing the intrinsic charge characteristics of graphene-based materials and their potential for environmental applications.

#### 2.2.5. XRF analysis method

To determine the oxide compositions of the soil samples, X-ray fluorescence (XRF) spectroscopy was employed in accordance with standardized procedures commonly used in geotechnical and environmental analysis. Prior to testing, the soil samples were thoroughly air-dried at room temperature until a constant weight was achieved. The dried samples were then finely ground and passed through a 200-mesh (75 μm) sieve to ensure uniform particle size and homogeneity, which are essential for accurate XRF analysis. The prepared pellets were loaded into the XRF spectrometer's sample holder for analysis. During the measurement, the samples were irradiated with primary X-rays, causing the emission of characteristic fluorescent X-rays from the oxides present in the soil. The intensities of these emitted X-rays were detected and quantified to determine the concentrations of various oxides. The analysis focused on major and minor oxides commonly found in soils, such as silicon dioxide (SiO_2_), aluminum oxide (Al_2_O_3_), iron oxide (Fe_2_O_3_), calcium oxide (CaO), magnesium oxide (MgO), potassium oxide (K_2_O), and sodium oxide (Na_2_O), among others.

#### 2.2.6. SEM analysis method

A scanning electron microscopy (SEM) analysis was conducted to examine the alterations in the microscale structural features of lead-contaminated loess before and after treatment. For this purpose, a Zeiss Gemini Sigma 300 scanning electron microscope (Oberkochen, Germany) was employed to observe particle morphology and inter-particle bonding within the soil matrix. The procedures for microstructural characterization in this study were refined based on the methodologies established in the works of Xu *et al.*,^36,37^ He *et al.*,^6^ and Hou *et al.*^38^

## 3. Results and discussion

### 3.1. Remediation of lead-contaminated loess using MICP

#### 3.1.1. Physical properties of lead-contaminated loess remediated by micp

**Fig. 7:**
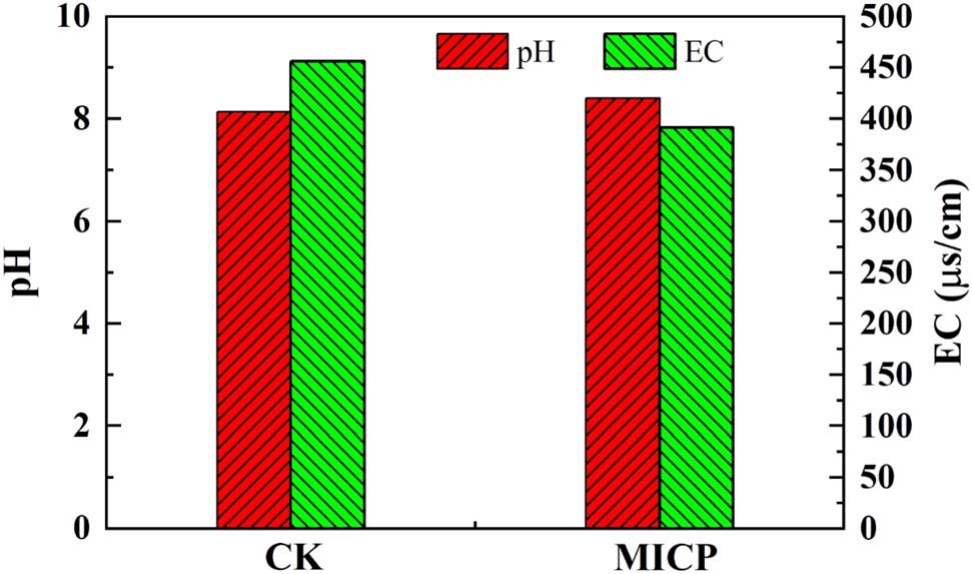
Distribution of lead chemical fractions in artificially contaminated loess.

Compared with the lead-contaminated loess in the control group (CK), the MICP solidification treatment significantly altered the chemical environment of the soil solution, as evidenced by an increase in soil pH and a marked reduction in electrical conductivity (EC), as shown in Fig. 7. These changes are primarily attributed to the alkalization reactions and ion immobilization effects induced during the MICP process. During MICP, urea hydrolysis occurs rapidly under the catalysis of urease-producing bacteria, generating large quantities of ammonia (NH_3_) and bicarbonate ions (HCO_3_^−^). The dissolution and equilibrium of ammonia in the pore water lead to a rapid increase in soil solution pH. Simultaneously, the bicarbonate ions react with calcium ions (Ca^2+^) to form calcium carbonate (CaCO_3_) precipitates, while the continuous presence of ammonium (NH_4_^+^) and dissolved ammonia maintains the alkaline conditions within the soil solution. In addition, the substantial precipitation of calcium carbonate effectively depletes Ca^2+^ and CO_3_^2−^ ions from the solution. The volatilization of ammonia under high-pH conditions further reduces the ionic concentration, resulting in a significant decline in electrical conductivity. The elevated pH observed during MICP not only stems from the alkalizing products of urea hydrolysis but also promotes the precipitation and immobilization of heavy metal ions. Under such alkaline conditions, lead ions (Pb^2+^) readily react with carbonate and hydroxide ions to form low-solubility lead precipitates, such as lead carbonate (PbCO_3_) or lead hydroxide (Pb(OH)_2_), thereby reducing the activity and mobility of heavy metals within the pore water. This passivation of heavy metals not only contributes to the reduction of soil solution conductivity but also enhances the microstructural stability of the solidified soil matrix. More importantly, the concurrent precipitation of calcium carbonate during the MICP process fills soil pores, markedly improving the microstructure by reducing pore connectivity. This structural densification effectively restricts the diffusion and transport of moisture and ions, serving as an additional mechanism responsible for the observed decrease in electrical conductivity.

#### 3.1.2. UCS of lead-contaminated loess remediated by MICP

**Fig. 8:**
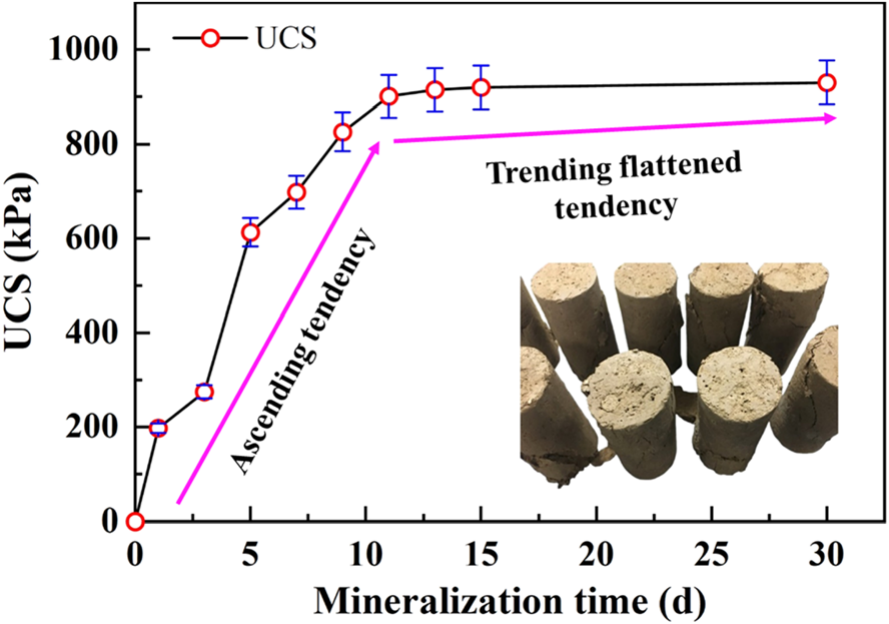
Relationship between unconfined compressive strength and mineralization time of specimens.

The variation of unconfined compressive strength (UCS) of the specimens with mineralization time is illustrated in Fig. 8. Visual observations of the mineralized samples reveal that the initially loose, lead-contaminated loess became structurally consolidated after mineralization. As the mineralization process progressed, the UCS of the MICP-treated specimens increased steadily. During the initial stage (1 to 3 days), the UCS exhibited a rapid rise, followed by a slower growth phase between 9 and 12 days. Beyond 9 days, the strength increment tended to stabilize, indicating that the mineralization reaction had approached equilibrium. According to the standards set by the U.S. Environmental Protection Agency, the UCS of solidified/stabilized waste for landfill disposal should not be less than 0.35 MPa. In this study, only the specimens mineralized for 1 to 3 days failed to meet this requirement. Specimens mineralized for 5 days or longer consistently exceeded the threshold, demonstrating sufficient mechanical stability. Notably, the UCS reached 826.5 kPa after 9 days of mineralization, indicating an optimal curing period. Therefore, a 9 days mineralization time was selected for subsequent stabilization tests.

#### 3.1.3. Leaching of lead-contaminated loess remediated by MICP

**Fig. 9:**
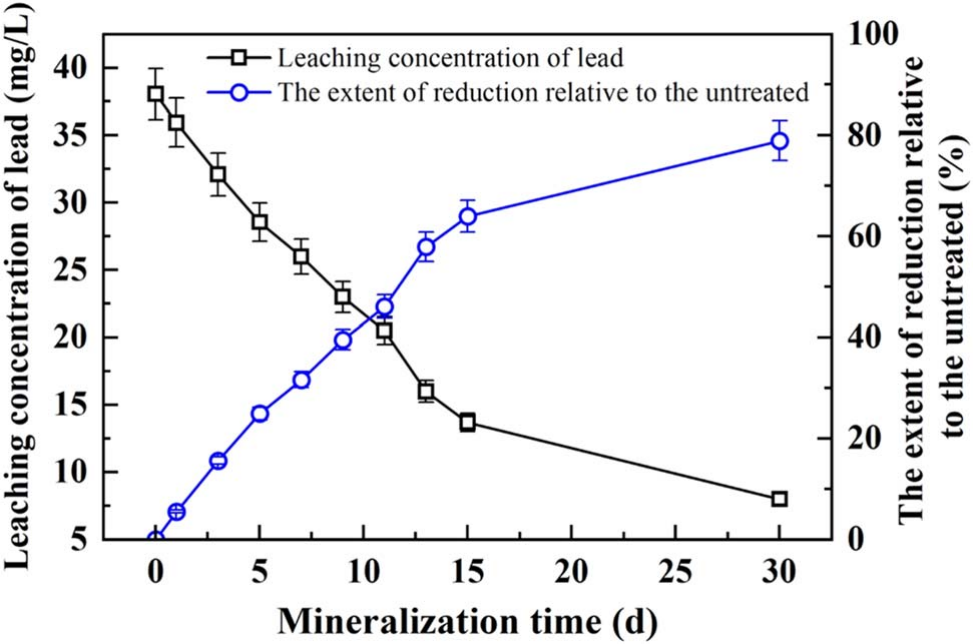
Relationship between leaching concentration of Pb and mineralization time of specimens.

The variation in the leaching concentration of lead in the solidified specimens of contaminated soil with increasing mineralization time is illustrated in Fig. 9. As the mineralization duration progressed, the leaching concentration of lead gradually decreased, with the reduction becoming more pronounced over time. During the initial stage, from day 1 to day 9, the decrease in lead concentration was relatively modest. However, after day 9, a more substantial decline was observed. When the mineralization time reached 9 days, the leaching concentration of lead dropped to 23.0 mg L^−1^, representing a 39.55% reduction compared with the untreated sample, which exhibited a concentration of 38.05 mg L^−1^.

### 3.2. Remediation of lead-contaminated loess using MICP and graphene-based materials

#### 3.2.1. Relationship between UCS and graphene material content

**Fig. 10:**
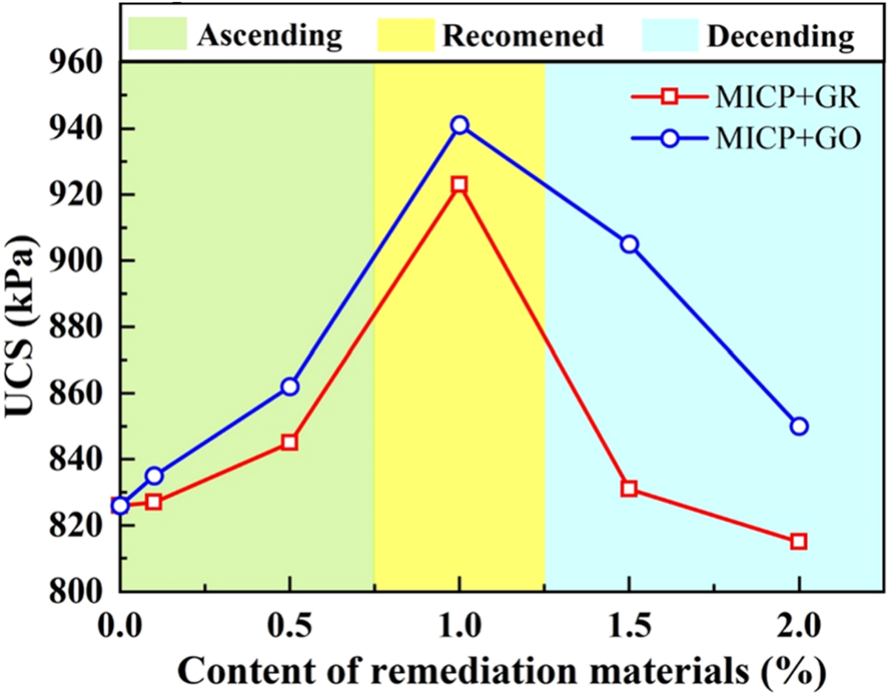
Relationship between unconfined compressive strength and content of different remediation materials.

Fig. 10 illustrates the variation trends in UCS of MICP-treated lead-contaminated loess specimens under different dosages of graphene-based adsorptive materials (GR and GO). Overall, compared to specimens treated solely with MICP, those subjected to combined treatment with adsorptive materials exhibited noticeably higher mechanical strength, indicating that the incorporation of graphene-based materials plays a positive role in enhancing soil structural stability. As shown in the Fig. 10, the UCS of the specimens first increases and then decreases with increasing GR or GO content. At lower dosage levels (≤1.0%), the addition of adsorptive materials effectively improved the distribution and homogeneity of the MICP-induced carbonate precipitates, while simultaneously optimizing the pore structure of the soil matrix, thereby significantly enhancing compressive performance. When the GR content reached 1.0%, the UCS peaked at 923 kPa. A further increase was observed with GO, where the UCS reached 941 kPa at the same 1.0% dosage, demonstrating a superior reinforcement effect. Notably, at 1.0% GO content, the UCS approached 950 kPa, representing an approximate 11.7% strength improvement compared to MICP treatment alone without GO addition.

However, when the dosage of adsorptive materials was further increased to 1.5% and 2.0%, a decreasing trend in UCS was observed. This decline may be attributed to particle agglomeration and pore-blocking effects caused by excessive amounts of adsorptive materials. High dosages disrupt the uniform distribution of calcium carbonate precipitates within the soil, resulting in localized structural defects that diminish the load-bearing capacity of the specimens. Although the UCS values at higher dosages remained generally higher than those of specimens treated solely with MICP, the mechanical enhancement effect was notably reduced. In summary, under the experimental conditions of this study, the optimal mechanical performance was achieved at a GO dosage of 1.0%, which maximized soil strength while avoiding the detrimental effects of overdosage. Thus, a 1.0% GO addition is recommended as the optimal proportion for combined remediation with MICP and adsorptive materials in this context.

#### 3.2.2. Relationship between leaching of lead and graphene material content

**Fig. 11:**
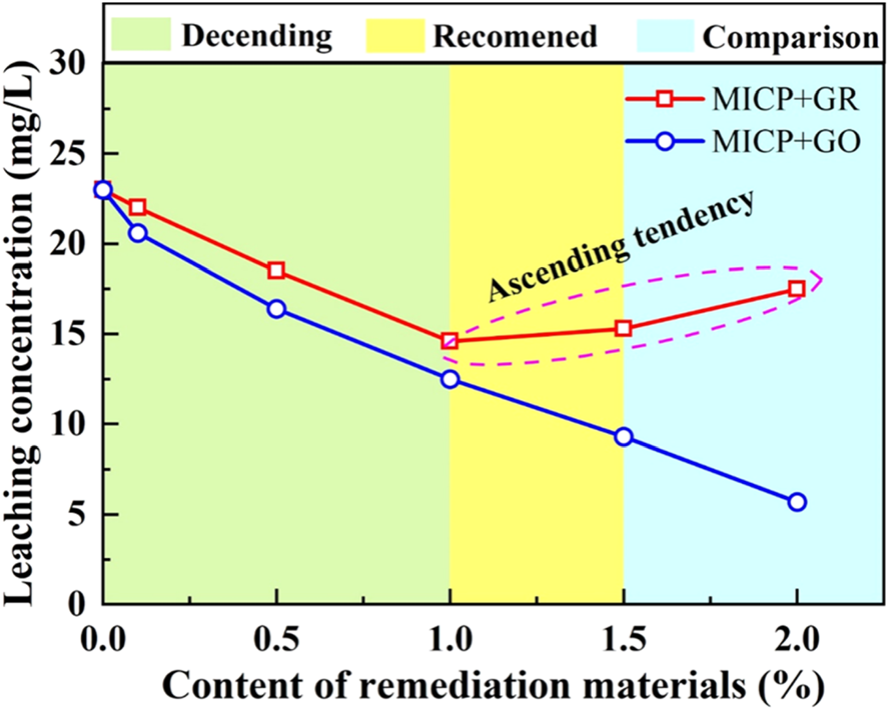
Relationship between leaching concentration of lead and content of different remediation materials.

Following the completion of UCS testing, all specimens were collected, labeled, and sealed in individual polyethylene bags for subsequent analysis of lead (Pb) leaching concentrations. Fig. 11 presents the variation in lead leaching concentrations under different dosages of graphene-based adsorptive materials (GR and GO). Overall, an increasing proportion of adsorptive material resulted in a consistent decline in lead leaching concentrations, demonstrating the synergistic effect of graphene materials in enhancing the immobilization performance of the MICP solidification process. Specifically, as the GR dosage increased from 0.1% to 1.0%, the leaching concentration of lead gradually decreased, reaching a minimum of 14.6 mg L^−1^ at 1.0% dosage. This represents a 61.63% reduction compared with the untreated sample (38.05 mg L^−1^), markedly surpassing the maximum reduction achieved by MICP alone (39.56%), with an additional immobilization enhancement of approximately 22.07%. However, when the GR content exceeded 1.0%, the lead concentration began to rise again, indicating that excessive GR may lead to structural heterogeneity or pore blockage within the soil matrix, hindering the formation of effective precipitates and thus diminishing the heavy metal immobilization efficiency. This phenomenon coincided with a decline in UCS, suggesting that overdosing GR negatively impacts both mechanical and environmental performance.

In contrast, GO exhibited superior performance throughout the tests. With increasing GO content from 0.1% to 1.0%, the lead leaching concentration decreased sharply. Beyond a 1.0% dosage, the reduction plateaued, with minimal further changes. Notably, at identical dosage levels, specimens treated with GO consistently displayed lower lead leaching concentrations than those treated with GR. At the optimal 1.0% dosage, the GO-treated specimens not only achieved the highest UCS but also reached the lowest lead leaching concentration, demonstrating the best balance between mechanical strength and environmental safety. In summary, GO outperformed GR as an adsorptive additive in the combined MICP remediation process. A 1.0% GO dosage emerged as the optimal formulation under the tested conditions, effectively enhancing soil strength while substantially reducing the environmental risk associated with lead leaching.

The effect of GR and GO dosage on Pb(ii) immobilization followed a nonlinear trend. As shown in Fig. 11, increasing the GR or GO content up to 1.0% significantly reduced the leaching concentration of lead in MICP-treated loess, with a maximum reduction of 61.63% and 65.75% for GR and GO respectively. This improvement is attributed to the high surface area and negative surface charge of the materials, which promote the chemisorption and electrostatic binding of Pb^2+^. GO, in particular, contains abundant –COOH and –OH groups, enabling stronger complexation with heavy metals.^34,35^ However, at higher dosages (1.5% and 2.0%), the immobilization efficiency slightly declined, possibly due to particle agglomeration and pore blockage that hinder the uniform distribution of calcium carbonate and reduce the accessibility of active adsorption sites. These findings suggest that an optimal additive concentration (1.0% GO) is critical to balancing microstructural enhancement and chemical immobilization of Pb(ii).

### 3.3. Mechanism of combined MICP and adsorptive material remediation for lead-contaminated loess

#### 3.3.1. Interaction mechanism between MICP and lead ions

**Fig. 12:**
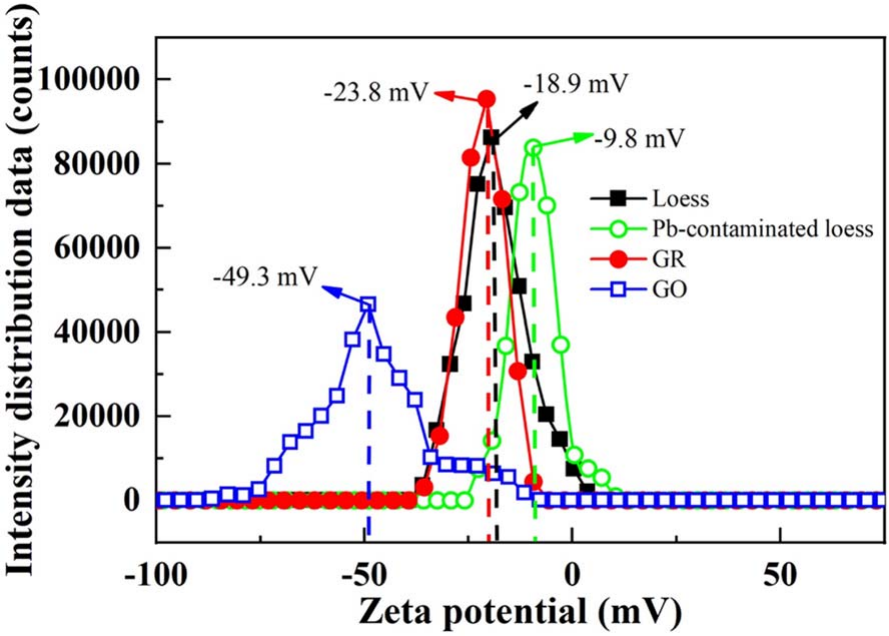
Zeta potential of different materials.

The remediation mechanism of lead-contaminated loess using MICP primarily involves a combination of microbial mineralization, heavy metal ion adsorption, and carbonate precipitation. Initially, natural loess particles exhibit a certain degree of negative surface charge, with a measured zeta potential of −18.9 mV (see Fig. 12). However, under lead contamination, Pb^2+^ ions interact with active surface sites on the soil particles through charge neutralization and complexation, leading to a notable increase in zeta potential to −9.8 mV. This shift reflects an enhanced surface charge shielding effect, reducing electrostatic repulsion between particles, promoting particle aggregation, altering pore structure, and ultimately affecting the soil's mechanical properties and transport behavior.

During the MICP process, urease-producing bacteria such as *Sporosarcina pasteurii* catalyze urea hydrolysis, generating large amounts of carbonate ions (CO_3_^2−^) and ammonium ions (NH_4_^+^).^49–51^ The carbonate ions then react with calcium ions (Ca^2+^) to form calcium carbonate (CaCO_3_) precipitates. Notably, bacterial cell walls are rich in negatively charged functional groups such as carboxyl and hydroxyl groups, which enable them to adsorb Pb^2+^ ions from the solution during the MICP process. This facilitates the nucleation of heavy metal carbonate precipitates around bacterial cells, promoting the formation of lead carbonate (PbCO_3_) microcrystals and enhancing the immobilization and stabilization of lead ions. Given the relatively low concentration of Pb^2+^ compared to Ca^2+^, the dominant mineral phases remain calcite (CaCO_3_) and vaterite, with lead carbonate present only in trace amounts through co-precipitation. Nevertheless, this process effectively reduces the activity of Pb^2+^ in the pore solution, improving the environmental stability of the soil.

Integrating the zeta potential results, this study further clarifies the mechanism of adsorptive materials in the combined remediation process. Both graphene (GR) and graphene oxide (GO) exhibit pronounced negative surface charges, with zeta potentials of −23.8 mV and −49.3 mV, respectively—far more negative than those of natural or lead-contaminated loess. The incorporation of these materials not only enhances the adsorption and immobilization of Pb^2+^ through surface charge effects but also improves the uniformity and dispersion of mineralization products, preventing crystal agglomeration and pore blockage during the precipitation of calcium carbonate. Among them, GO demonstrates superior performance due to its stronger negative charge and higher density of oxygen-containing functional groups, resulting in more effective lead immobilization and greater enhancement of soil mechanical strength.

#### 3.3.2. Interaction mechanism between MICP and adsorptive materials

The enhanced remediation of lead-contaminated loess through the combined application of MICP and graphene-based adsorptive materials is primarily attributed to a multi-faceted coupling mechanism involving microbial-induced mineralization, adsorptive interactions, and heavy metal immobilization.^52–54^ Analysis of the oxide composition revealed a distinct trend across natural loess (loess), lead-contaminated loess (LCL), MICP-treated specimens (LCL modified by MICP), and specimens subjected to combined treatment with MICP and graphene-based materials (LCL modified by MICP + GR/GO). As remediation intensity increased, the contents of major oxides such as SiO_2_, Al_2_O_3_, and MgO progressively decreased, while PbO content showed a continuous rise. This pattern reflects the enhanced immobilization of Pb^2+^ ions during treatment, accompanied by their gradual transformation from soluble forms to stable mineral phases.

The underlying mechanism can be summarized by several key processes. Initially, in lead-contaminated loess, the infiltration of Pb^2+^ ions causes a significant increase in soil zeta potential—from −18.9 mV in natural loess to −9.8 mV—indicating intensified surface charge shielding and reduced inter-particle adsorption capacity, which in turn elevates the risk of heavy metal migration. During the MICP process, urease-producing bacteria catalyze urea hydrolysis, generating abundant CO_3_^2−^ ions that react with Ca^2+^ to form calcium carbonate (CaCO_3_) precipitates. Simultaneously, the negatively charged bacterial surfaces adsorb Pb^2+^ ions, promoting the localized formation of lead carbonate (PbCO_3_) around bacterial cells and effectively reducing the mobility of Pb^2+^ within the soil matrix.

Building upon this, the incorporation of adsorptive materials further amplifies the immobilization of heavy metals. Both GR and GO possess strongly negative surface charges, with zeta potentials of −23.8 mV and −49.3 mV, respectively. GO, in particular, exhibits superior performance due to its abundant hydroxyl and carboxyl functional groups, high specific surface area, and elevated chemical reactivity. These functional groups facilitate the chelation and physical-chemical adsorption of Pb^2+^ ions, providing an initial stabilization mechanism. Moreover, the nanopores and layered structures of these materials offer nucleation sites for subsequent heavy metal carbonate precipitation. Specifically, hydroxyl and carboxyl groups in GO capture Pb^2+^ ions, creating localized enrichment zones within pores or on surfaces that promote lead carbonate deposition and stabilization. Concurrently, these materials mitigate excessive agglomeration of calcium carbonate, ensuring a more uniform distribution of mineralization products and enhancing soil compaction. Following mineralization, lead is immobilized within the soil through three primary pathways:

(i) precipitation as lead carbonate on microbial surfaces and within soil pores;

(ii) chemical adsorption or chelation onto the surfaces and within the pores of GR/GO materials;

(iii) physical entrapment within calcium carbonate crystals or the nanoporous structures of adsorptive materials, forming a composite immobilization mechanism integrating physical encapsulation, chemical chelation, and mineral co-precipitation.

**Fig. 13:**
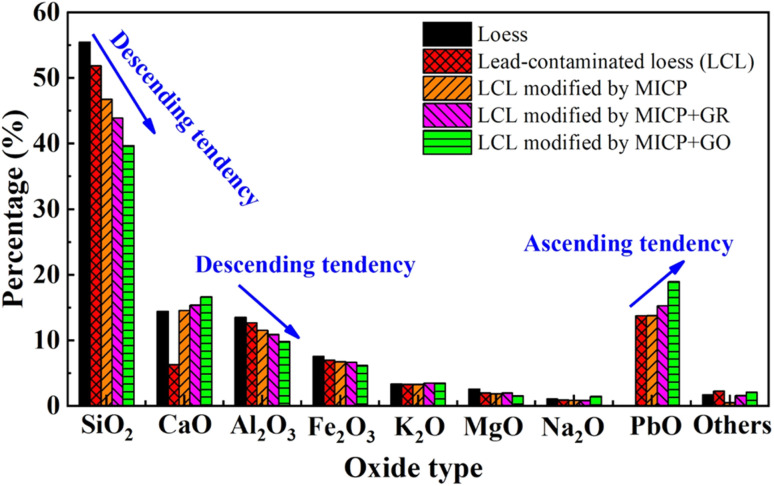
Variation patterns of oxide composition under different remediation conditions.

In summary, while MICP primarily drives the mineralization and precipitation of heavy metals as stable carbonates, the introduction of adsorptive materials synergistically enhances Pb^2+^ immobilization through surface charge modulation, chemical chelation, and structural confinement effects. This combined mechanism not only explains the observed increase in PbO content in oxide analyses but also elucidates the microstructural processes and environmental benefits underlying the joint remediation approach involving MICP and graphene-based materials (Fig. 13).

#### 3.3.3. Mechanism of strength enhancement and lead immobilization by adsorptive materials

**Fig. 14:**
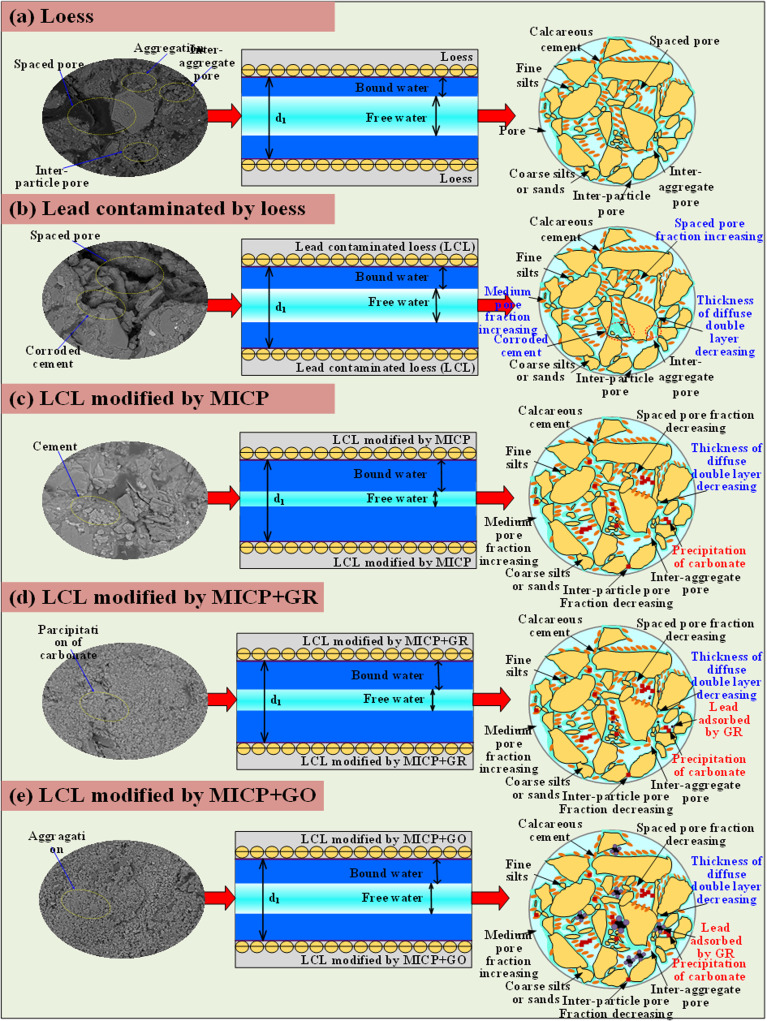
Schematical illustration of the (a) loess microstructure exposed to: (b) lead, (c) MICP technology and (d and e) MICP technology with adsorption materials.

As shown in Fig. 14, the microstructural characteristics and physicochemical changes of the five tested specimens demonstrate a clear and progressive evolution throughout the remediation process. The SEM images intuitively reflect the transformation of soil structures under different treatments. Natural loess exhibits a loosely packed microstructure with irregular particle arrangements and prominent intergranular pores (Fig. 14a). Upon lead contamination, the soil particles undergo significant surface modifications. The infiltration of Pb^2+^ ions leads to notable electrostatic neutralization and charge shielding, as evidenced by the sharp increase in zeta potential from −18.9 mV to −9.8 mV, weakening electrostatic repulsion among particles and promoting aggregation. This results in the collapse of pore structures and an increase in heavy metal migration risk (Fig. 14b). With the progression of MICP treatment, the SEM images reveal substantial calcium carbonate (CaCO_3_) precipitation, which bridges and binds soil particles through microbial-induced mineralization (Fig. 14c). The double-layer thickness analysis further corroborates this observation, indicating a reduction in electrostatic repulsion and enhanced inter-particle bonding as the MICP process proceeds. The formation of calcite and vaterite fills soil voids, consolidates the particle framework, and significantly improves soil compactness and mechanical stability. Following the incorporation of graphene-based adsorptive materials (Fig. 14d), especially GO, the soil microstructure becomes more densely packed and homogeneous (Fig. 14e). The SEM images demonstrate that the addition of these materials effectively mitigates excessive CaCO_3_ aggregation and promotes the formation of a well-dispersed, finely interlocked microstructure. The double-layer thickness decreases even further, particularly in the GO-treated specimens, reflecting intensified charge interactions and improved soil stability. The highly negative zeta potentials of GR (−23.8 mV) and GO (−49.3 mV) not only enhance the physical adsorption of Pb^2+^ ions but also regulate the distribution of carbonate precipitation, preventing localized clogging and ensuring uniform mineralization throughout the soil matrix.

The schematic diagrams in Fig. 14 further illustrate the coupled remediation mechanism. Pb^2+^ ions are initially adsorbed onto the functional surfaces of GR and GO *via* electrostatic attraction and chemical complexation. These sites subsequently act as nucleation centers for carbonate precipitation, facilitating the formation of stable lead carbonate (PbCO_3_) and calcium carbonate phases. GO, in particular, provides a higher density of nucleation sites due to its abundant oxygen-containing functional groups and layered structure, which not only strengthens heavy metal immobilization but also promotes pore filling and structural densification. Ultimately, lead ions in the treated soils are immobilized through three pathways: precipitation as lead carbonate within soil pores and around bacterial surfaces, chemical adsorption or chelation on GR/GO surfaces, and physical entrapment within calcium carbonate crystals or the nanoporous structures of the adsorptive materials. This combined immobilization mechanism explains the observed increase in PbO content across the oxide analyses and accounts for the simultaneous improvements in mechanical strength and environmental stability. In summary, the SEM observations, electrochemical analyses, and structural schematics jointly demonstrate that the remediation of lead-contaminated loess through MICP, particularly when enhanced by GO, results in a dense, stable, and highly immobilized soil matrix. The process not only repairs structural damage caused by lead contamination but also establishes a synergistic stabilization framework, wherein microbial mineralization and advanced adsorption materials work in concert to achieve optimal mechanical and environmental outcomes.

In details, the mechanism underlying the enhanced immobilization of Pb^2+^ in loess through the combined application of microbial-induced carbonate precipitation (MICP) and graphene-based adsorptive materials (GR and GO) was further elucidated. The process is governed by a synergistic interaction between biochemical mineralization, physicochemical adsorption, and microstructural modification. During MICP treatment, urease-producing bacteria catalyze the hydrolysis of urea into ammonium and carbonate ions. The latter subsequently reacts with calcium ions to precipitate calcium carbonate, which serves not only as a binding agent that bridges soil particles, but also as a matrix for heavy metal co-precipitation. Although Pb^2+^ has a lower thermodynamic propensity to form carbonate than Ca^2+^, it can be incorporated into the mineral phase either through surface adsorption onto calcite or *via* substitution within the crystal lattice, as reported in studies by Fu *et al.*^45^ and Tang *et al.*^49^ Beyond microbial mineralization, the introduction of graphene (GR) and particularly graphene oxide (GO) significantly improves the adsorption affinity for Pb^2+^. GO exhibits a high density of oxygen-containing functional groups (*e.g.*, –COOH, –OH, –C=O), which facilitate strong chemisorption of metal ions *via* complexation and electrostatic attraction. Zeta potential measurements confirm that the surface charge of GO (−49.3 mV) is considerably more negative than that of contaminated loess (−9.8 mV) (see Fig. 12), promoting selective binding of divalent cations and suppressing their mobility in pore water. This observation aligns with the findings of Kang *et al.*,^54^ who demonstrated that GO-modified hydrogel electrodes enhanced metal sequestration under electrokinetic conditions by providing abundant reactive sites.

Moreover, the functional surfaces of GO serve as heterogeneous nucleation sites for carbonate mineralization. The localized enrichment of Pb^2+^ on GO layers facilitates the formation of microcrystalline PbCO_3_ or Pb-substituted calcite, leading to spatially confined and more stable heavy metal retention. This nucleation behavior also reduces the risk of excessive CaCO_3_ agglomeration, improving mineral dispersion and pore filling efficiency. SEM analysis in this study supports this mechanism, revealing a denser and more continuous cementation matrix in the GO-added specimens (see Fig. 14). The subsequent enhancement in mechanical strength and reduction in Pb leachability provide strong evidence for the dual role of GO in both structural reinforcement and contaminant stabilization. In addition, the interaction between GR/GO and bacterial cell walls may influence the metabolic activity and spatial distribution of ureolytic bacteria. As suggested by Kumar *et al.*,^50^ the presence of nanoscale carbon materials can modify bacterial colonization patterns, thereby affecting the kinetics and uniformity of carbonate precipitation. While this biological modulation was not the main focus of the present study, it may offer an additional pathway for optimizing MICP–adsorbent synergy in future work. Taken together, the integrated immobilization mechanism involves chemical precipitation, surface complexation, ion exchange, and physical encapsulation, all of which are enhanced by the introduction of graphene-based materials. This combined approach demonstrates superior performance compared to MICP or adsorption alone, especially in low-permeability soils like loess where uniform treatment and multi-scale stabilization are critical.

In real contaminated soils, the presence of multiple heavy metals often complicates the MICP process due to differences in metal ion behavior. The selectivity of carbonate precipitation follows thermodynamic rules governed by the solubility product (*K*_sp_): PbCO_3_ and CuCO_3_ typically form earlier than ZnCO_3_ or CdCO_3_.^18^ Therefore, rational application of MICP under multi-metal scenarios requires prior identification of dominant contaminants, as well as adjustment of calcium ion concentration, pH, and bacterial activity to control the precipitation sequence. Additionally, adsorption-based selectivity on GO/GR surfaces can be leveraged to preferentially immobilize certain ions. Future work should develop predictive models incorporating competitive adsorption and precipitation kinetics to standardize the process for complex field environments. The combined MICP–graphene-based remediation system has potential for large-scale application because it is compatible with existing *in situ* soil treatment technologies such as injection wells and surface infiltration. MICP relies on the natural proliferation of ureolytic bacteria, and graphene-based additives can be co-delivered in suspension without significant modification to the process. Previous pilot-scale studies of MICP have demonstrated its feasibility in permeating low-permeability soils, suggesting that the approach can be adapted to large soil matrices. However, field implementation will require optimization to ensure the homogeneous distribution of microorganisms and additives, as well as cost and sustainability assessments. These aspects will be addressed in future work.

Although this study focused on Pb(ii) immobilization, the combined MICP–graphene-based treatment strategy is theoretically applicable to other common heavy metal ions in loess, such as Cu(ii), Cd(ii), and Zn(ii). The mechanisms governing their immobilization—carbonate precipitation, electrostatic adsorption, and surface complexation—are not ion-specific and can accommodate a range of divalent cations. However, differences in solubility product (*K*_sp_), ionic radius, and affinity to functional groups (*e.g.*, –COOH, –OH) may lead to variations in immobilization efficiency. For example, Cu^2+^ tends to co-precipitate with carbonate earlier than Zn^2+^, while Cd^2+^ shows relatively lower affinity to GO surfaces under neutral pH. Thus, future studies should explore the competitive and cooperative behaviors of multiple metal ions in complex contamination scenarios to fully validate the broader applicability of this approach in field conditions.

## 4. Conclusions

This study systematically investigated the coupled mechanism and remediation performance of MICP combined with graphene-based adsorptive materials for the treatment of lead-contaminated loess. Overall, the results and discussion lead to some main conclusions.

(1) MICP alone effectively improved both mechanical strength and environmental safety of lead-contaminated loess through microbially induced carbonate precipitation and microbial surface adsorption, forming a denser soil structure and reducing lead leaching concentrations by up to 39.56%.

(2) Graphene-based materials further enhanced the remediation performance by increasing soil strength and immobilizing Pb^2+^ more effectively. Both GR and GO improved the homogeneity of carbonate precipitation and soil compactness, while also reducing Pb^2+^ migration through surface adsorption and complexation. Among them, GO showed superior performance due to its stronger negative surface charge and higher functional group density.

(3) Optimal results were achieved with a GO dosage of 1.0%, which simultaneously maximized UCS (approximately 950 kPa) and minimized lead leaching concentration (14.6 mg L^−1^), far outperforming both MICP alone and MICP combined with GR.

(4) Microstructural analysis revealed a synergistic immobilization mechanism involving microbial carbonate precipitation, chemical adsorption, and physical encapsulation. The combined process reduced double-layer thickness, improved particle bonding, and facilitated the formation of lead carbonate precipitates within the soil matrix and adsorptive material pores.

## Author contributions

Xinwen Wang: investigation, formal analysis, writing – reviewing and editing. Shixu Zhang: conceptualization, methodology, resources, writing – original draft preparation. Ke Chen: writing – original draft preparation.

## Conflicts of interest

The authors declare that there is no conflict of interest regarding the publication of this paper.

## Data Availability

The data used to support the findings of this study are included within the article.
